# Understanding Feedback for Learners in Interprofessional Settings: A Scoping Review

**DOI:** 10.3390/ijerph191710732

**Published:** 2022-08-29

**Authors:** Varun Coelho, Andrew Scott, Elif Bilgic, Amy Keuhl, Matthew Sibbald

**Affiliations:** 1 Faculty of Health Sciences, McMaster University, Hamilton, ON L8S 4L8, Canada; 2 Department of Pediatrics, McMaster University, Hamilton, ON L8S 4L8, Canada; 3 Department of Medicine, McMaster University, Hamilton, ON L8S 4L8, Canada

**Keywords:** feedback, interprofessional, education, training

## Abstract

Background: Interprofessional feedback is becoming increasingly emphasized within health professions’ training programs. The objective of this scoping review is to determine what is known about how learners perceive and interact with feedback in an interprofessional context for learning. Methods: A search strategy was developed and conducted in Ovid MEDLINE. Title and abstract screening were performed by two reviewers independently. Next, full texts of selected articles were reviewed by one reviewer to determine the articles included in the review. Data extraction was performed to determine the articles’ study population, methodologies and outcomes relevant to the research objective. Results: Our analysis of the relevant outcomes yielded four key concepts: (1) issues with the feedback process and the need for training; (2) the perception of feedback providers, affecting how the feedback is utilized; (3) professions of the feedback providers, affecting the feedback process; and (4) learners’ own attitude toward feedback, affecting the feedback process. Conclusions: The learner’s perception of interprofessional feedback can be an obstacle in the feedback process. Training around interprofessional feedback should be included as part of interprofessional programs. Research is needed to explore how to address barriers in feedback interaction that stem from misguided perceptions of feedback providers’ professions.

## 1. Introduction

The need for interprofessional education in healthcare has been growing for many years. In a 2010 publication, the World Health Organization recognized “interprofessional collaboration in education and practice as an innovative strategy that will play an important role in mitigating the global health workforce crisis” [1]. The Canadian Interprofessional Health Collaborative (CIHC) has defined interprofessional collaboration as the “process of developing and maintaining effective interprofessional working relationships with learners, practitioners, patients/clients/families and communities to enable optimal health outcomes” [2]. The CIHC National Interprofessional Competency Framework identifies six competency domains that are necessary for interprofessional collaboration: interprofessional communication, patient/client/family/community-centered care, role clarification, team functioning, collaborative leadership, and interprofessional conflict resolution [2]. Competency in these domains is essential for effective interprofessional collaboration and must be taught to health-profession learners. Interprofessional teams in healthcare can be quite diverse and can include learners, nurses, physicians, physical therapists, pharmacists, technicians, rehabilitation specialists, social workers and other professions that assist in the care of the patient. Different clinical scenarios can involve different fields working together to provide the best possible care to the patient. Interprofessional communication between these professions, particularly in delivering feedback and receiving feedback, has been recognized as an essential competency [3].

Feedback is defined as information about a person’s performance that serves to improve his or her capabilities and promote positive development [4,5]. The main characteristic of feedback is to provide learners with information about their observed performance compared to the desired performance [4]. Giving and receiving feedback is of vital importance in learning [4]. It is an essential component of education and provides learners an opportunity to gain valuable insight into their performance. Receiving constructive and timely feedback can help reduce the gap between actual and desired performance [5,6]. Importantly, feedback has been shown to be essential in health education in terms of learning and improving one’s skills [4,7]. Interprofessional teamwork, which includes feedback interactions, is recognized as important for quality patient care [8]. Although the literature highlights the importance of interprofessional feedback, there is less known about the process in which interprofessional learners give and receive feedback. Feedback delivery is now being recognized as not a simple flow of criticism and comments from teacher to learner, but rather a more complex process [5,9,10]. Understanding the various dimensions to giving and receiving feedback is of importance for interprofessional education, so that interprofessional learners can truly benefit from the feedback process.

A preliminary search of different databases, conducted on Ovid MEDLINE, showed very few reviews of the literature on how learners give and receive feedback in an interprofessional setting. Understanding what the literature says about interprofessional feedback is particularly important for educators. Knowing what makes feedback effective can assist educators seeking to teach health-profession learners in interprofessional settings. Scoping reviews provide a unique opportunity to analyze the existing literature to identify key concepts; gaps in the research; and types and sources of evidence to inform practice, policymaking and research [11]. Thus, this review’s objective was to understand what is known about how undergraduate learners give and receive feedback in an interprofessional context for learning.

## 2. Materials and Methods

This scoping review was guided by using the Arksey and O’Malley’s framework [11]. Our protocol was primarily formed in accordance with the Preferred Reporting Items for Systematic reviews and Meta-Analyses extension for Scoping Reviews (PRISMA-ScR) [12] and was revised by the research team. A scoping review was chosen as this study’s methodology, as scoping reviews provide the opportunity to map out the literature. Interprofessional education is a broad topic; therefore, a scoping review was chosen to identify what is known on this topic.

### 2.1. Review Question

What is known about how health professions’ learners give and receive feedback in an interprofessional context for learning?

### 2.2. Search Strategy

A search strategy was planned with the assistance of a health-sciences librarian. Keywords and Medical Subject Headings were identified: education, medical education, undergraduate medical education, feedback, feedback literacy, operations research, interprofessional, interprofessional relations and additional terms. The specific search algorithm used can be found in Appendix A, Table A1. A search of Ovid MEDLINE was conducted in October of 2021 with all of these terms. All relevant citations were uploaded into Covidence [13], where duplicate references were identified and removed.

### 2.3. Eligibility Criteria

We included all studies where empirical data were generated. This included studies with experimental, quasi-experimental, observational (e.g., prospective cohort) and qualitative (e.g., grounded theory) methodologies. Narrative or opinion-based studies were excluded. This scoping review focused on health professions’ learners in interprofessional settings. Specifically, these learners must be part of a profession that involves interactions with other professions. There were no restrictions on professions of the study participants as long as they were healthcare related and included in an interprofessional context. Works from the literature that included preceptors in the study population were included if learners were involved in its study population as well. Any works from the literature that did not include health professions’ learners was excluded. Studies with a central focus on feedback were included in this review. These included studies that analyze perceptions of feedback, the utility of feedback and the giving of feedback. Studies that had feedback as a secondary outcome, where it was not the central focus of the paper, were excluded. This scoping review analyzed papers published in English from 2001 to 2021 in any country. Geographic location was not a factor in inclusion. Additionally, this review was not limited by any racial, gender or socioeconomic demographics and considered all interprofessional learners.

### 2.4. Screening

Following the upload to Covidence, two independent reviewers screened articles by title and abstract to determine if they should be included in the full-text review. The reviewers met after their initial screening to resolve any conflicts. A full-text review of the remaining articles followed wherein the articles were assessed against the inclusion criteria. Reasons as to why any articles were excluded were recorded.

### 2.5. Data Extraction

Data were extracted from all papers selected for the scoping review, and this was performed by a single reviewer. A data-extraction spreadsheet was developed by reviewers and included data items with the following headings: “study details”, which included details on author(s), date and country; “study objective”; “study population”; “methodology”; and “relevant outcomes”. While no specific appraisal tool was used to assess selected articles, the quality of articles was assessed by recording limitations found in the studies. Outcomes were deemed relevant if they answered the review question. The data were analyzed by a single reviewer who filled in the data-extraction table. The completed table can be seen in Table A2 in Appendix A. After data extraction, the relevant outcomes of the papers were analyzed to find key concepts among the articles. This was performed by finding and recording common themes amongst the articles.

## 3. Results

**Figure 1:**
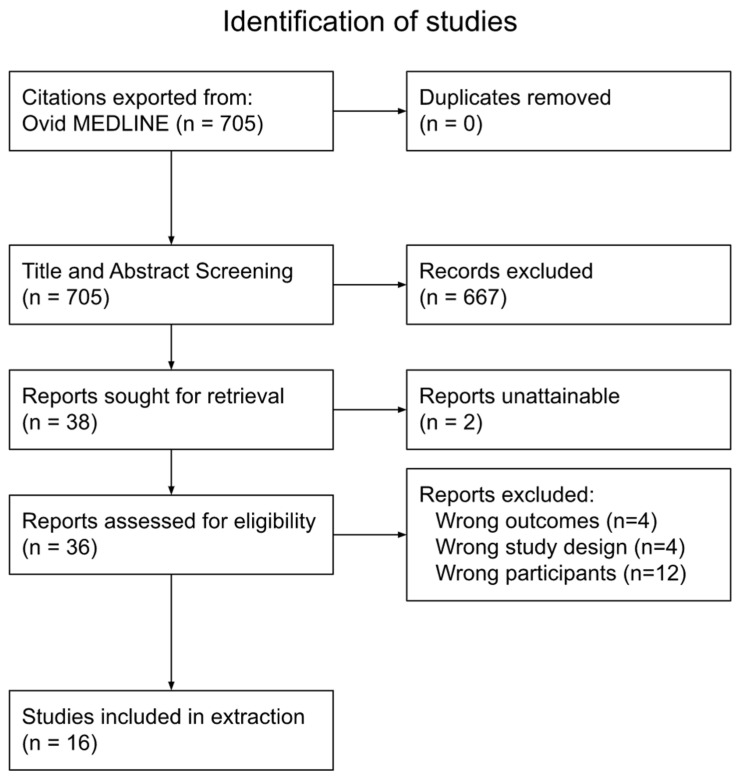
PRISMA-ScR diagram.

The search of Ovid MEDLINE yielded 705 articles. After the first screening, 667 articles were excluded. This left 38 articles, two of which had reports that were unattainable. Therefore, 36 articles were assessed for eligibility through a full-text review. This resulted in 20 articles being excluded, as they did not adhere to the eligibility criteria. Specifically, 12 studies were excluded for wrong outcomes, 4 were excluded for wrong participants and 4 were excluded for wrong study design. This left 16 articles for data extraction. A summary of the process is shown in Figure 1.

### 3.1. Literature Characteristics

Of the 16 articles, 11 were published between 2016 to 2021. The other five articles were published between 2007 and 2013. All articles had more than one author, and they all took place in North America or Europe, except for one article which did not have a specific country of focus. The methodologies of the studies varied. Nine articles used qualitative approaches, six used quantitative approaches and one used mixed-method approaches. In terms of the study population, medical residents were a common study population, with five studies exclusively focusing on them. Additionally, three studies focused on medical/healthcare students; one study focused on dental students and another focused on veterinary medical students. The other articles (*n* = 5) contained multiple health professions in its study population. These professions include medical residents, physicians (supervisory physicians and general practitioners), nurses, nutritionists, psychologists, rehabilitation therapists, social workers, pharmacists, pharmacy students and medical students. Of these studies, residents were present in three articles, and nurses were present in three articles, as well. Specific details on articles’ details, population, methodology and outcomes can be found in Table A2 in Appendix A.

### 3.2. Key Concepts

#### 3.2.1. Issues with the Feedback Process and the Need for Training

Six studies identified a need for training on the feedback process to address issues in the giving and receiving of feedback. Issues related to giving feedback include feedback not being delivered on time, the overabundance of feedback evaluations and feedback being too vague [14]. One article showed that medical residents considered feedback from all other professions as suboptimal [15]. Health-professional learners indicated that feedback which promotes learners’ professional development needs to be timely, constructive, encouraging and focused on ways to improve [16]. Additionally, the process of giving feedback can be moderately challenging, as well, with one study giving a survey to interprofessional learners which asked them how challenging the process was of giving feedback to their interprofessional peers [17]. One study that had healthcare students undergo a feedback-literacy program had positive effects [18]. For example, students realized they could actively seek feedback instead of waiting for it and that they could engage in the feedback process and make plans of improvement. This relates to another article which showed that medical students’ own initiative led them to find feedback more instructive [19]. From these articles, it becomes clear that there is a need for training for health-professional learners in how to approach the feedback process.

#### 3.2.2. Perception of the Feedback Provider Affects How the Feedback Is Utilized

Seven articles suggested that a learner’s perception of the person providing the feedback affects how learners react to the feedback. In one study that analyzed learners’ perceptions of feedback regarding the learner–educator relationship, it found that attitudes, perceptions, relationships and teacher attributes affected feedback-seeking behavior [20]. Another study found that feedback-seeking behavior is influenced by the leadership styles of the feedback provider [21]. It found, through a review of the literature, that learners tended to seek feedback from their supervisor if they viewed them as a transformational leader. This supportive nature of teachers has also been shown in a separate article that gave a questionnaire to residents to assess predictor, mediator and outcome variables [22]. This article found that supportive leadership led residents to perceive more feedback benefits and fewer costs. Another study found that interpersonal factors, such as the relationship between the learner and the feedback provider, significantly influenced feedback-seeking behavior [23]. In another study, residents were found to actively contemplate the feedback they received based on their judgments of the feedback provider’s clinical credibility and their relationship with that feedback provider [24]. Similarly, another article found that identity and hierarchy is a significant theme in the feedback process, with social identities being the source of these interprofessional hierarchies [25]. In that article, residents valued interprofessional feedback and had a positive attitude toward it. However, regarding feedback from supervising physicians (an intra-professional identity to residents), the identities of “trainee” and “supervisor” became more highlighted as impeding receptiveness of feedback. This relates to another article where dental students were asked to evaluate their experience with feedback through a questionnaire and found that students rated faculty-led feedback higher than peer-led feedback [26]. These articles indicate that the way in which the learners view the feedback provider significantly influences the way that feedback is received.

#### 3.2.3. Professions of the Feedback Provider Affect the Feedback Process

The profession of the feedback provider can play a significant role in the feedback process. A study with a randomized control trial, with pediatric residents, highlighted the importance of multisource feedback [27]. Interestingly it showed that nurse ratings differed significantly than ratings from parents of the patients. This can indicate that the role or profession of the feedback provider matters in terms of the context of the feedback. Another concept that was found was that learners tend to value feedback more if it comes from their own profession. Three articles highlighted this theme, although one article seemingly contradicts it. One study found that there were significant interactions between the labelled profession of the feedback provider and the feedback recipient [28]. This means that nurses rated feedback that were labelled to be from nurses higher than feedback that was labelled to be from physicians, and vice versa. Similarly, two other studies showed that residents were found to value feedback from physicians more so than other health professionals [29] and were more likely to act on said feedback [15]. Residents preferred feedback from their in-group (physicians), as they felt physicians had better understanding of residents’ expectations and often focused on feedback that residents perceived as more valuable [15]. However, one article stated in its outcomes that the profession of the feedback provider had no main effect; there were no significant interactions between the professions of the feedback recipient and the feedback provider [17]. This article is contradictory to what the other articles were concluding; however, the article did find that the profession of the feedback recipient affected how feedback was rated, with some professions giving higher ratings than others. In conclusion, learners may value feedback from their own professions; however, more research may be needed to confirm this notion.

#### 3.2.4. Learners’ Own Attitude toward Feedback Can Affect the Feedback Process

A final concept that emerged from the data was the effect of the attitude of the learner. Three articles highlighted this concept. Two articles showed that learners with learning-goal orientations had positive interactions with feedback [21,23]. Learning-goal orientations, as described by the articles, is the desire to develop oneself by improving one’s skills and competence and mastering new situations [21,23]. One article found that there is higher value in feedback when oriented learners have learning goals because these learners view their abilities as something that can be improved over time [21]. These learners also view failure as a way to increase effort, making them less afraid of negative feedback. Another study found that residents who perceived benefits from feedback were found to report a higher frequency of feedback inquiry and monitoring (taking in self-relevant information from the environment by observation of others) [22]. These articles show that the attitude of the learner plays a prominent role in how they receive feedback.

## 4. Discussion

### 4.1. Summary of Main Findings

This scoping review focused on reviewing the scientific literature to discover what is known about how health-professional learners give and receive feedback in an interprofessional setting for learning. The literature reports used a variety of methodologies, with most using qualitative methods. Additionally, residents were found to be the most common study population. An analysis on the outcomes of the study yielded four key concepts: (1) issues with the feedback process and the need for training, (2) the perception of the feedback provider affects how feedback is utilized, (3) the professions of the feedback provider affect the feedback process and (4) the learners’ own attitude toward feedback can affect the feedback process.

### 4.2. Discussion of Findings

The key concepts bring to light many important elements on how learners give and receive feedback in the interprofessional environment. First, in regard to the issues in the feedback process, the findings show that training on the feedback process is needed. The articles from the first key concept indicated that feedback across professions can be suboptimal if it is not timely, specific, constructive and encouraging [14,15,16]. The article that used a literacy program for teaching feedback showed that these barriers can be partly addressed through education [17]. Additional evidence also suggests that improving the feedback literacy of students and staff could promote effective feedback practices and address some of the barriers [30,31]. This is important, as the type of feedback and the way it is given can have a significant impact on the feedback recipient. In particular, feedback has been shown to be more effective when it specifically focuses on a certain task and how to improve upon it, rather than feedback that just praises or punishes behavior [6]. Feedback, if not delivered properly, can even have negative effects on learners [9], thus highlighting the importance for improving it.

Additionally, the findings of the scoping review also show that the attitude of learners toward feedback can greatly affect how it is used. Learners with learning-goal orientations were mentioned in three articles of the scope [21,22,23]. Thus, teaching learners how to orient themselves in a way that is focused on improving oneself can potentially improve their competence. Therefore, it can be interpreted from the findings that feedback literacy needs to be implemented for both feedback providers and feedback recipients in order for feedback to be effective for learning.

Interprofessional teams can be quite diverse and include multiple professions collaborating to provide the best possible care to the patient. This diversity in medicine can be a potential strength in healthcare, as it allows the perspectives from multiple fields to be brought into consideration. However, one must understand the various group dynamics that come into play in an interprofessional environment. Perception of the feedback provider, including the perception of their profession, seems to play a significant role in how interprofessional feedback is used and interpreted. Studies showed that in-group and out-group biases can have important interactions on feedback in interprofessional settings. For instance, nurses will value feedback from nurses more so than feedback from physicians and vice versa [28]. These findings are not entirely surprising, as social-identity theory predicts that individuals will preferentially value perspectives from the group they identify with [32]. This can potentially be a negative aspect, as an interprofessional team can lose its inherent value by members of the team only valuing perspectives from their own profession. Group processes in medicine through the lens of social-identity theory can bring forth tensions in interprofessional teams which can affect the utility of feedback [33].

### 4.3. Limitations

The search of only one database, Ovid MEDLINE, is a limitation, as it may have restricted the number of articles that were found. Searching more databases may have provided more studies or a greater variety of study population. Additionally, the terms used for searching Ovid MEDLINE may not have been comprehensive enough and could have limited the articles found. Furthermore, all of the literature came from North America and Europe. This can potentially limit the scope of interprofessional education, as culture could play a role in interprofessional feedback. Additionally, there is a lack of confirmatory work in this scoping review. Having replication studies could help confirm the key concepts that were found.

## 5. Conclusions

Feedback among health-profession learners in an interprofessional context consists of many factors and is immensely important in health professions’ education, especially considering the growing need for interprofessional collaboration. This scoping review highlights important findings about how learners give and receive feedback in an interprofessional setting for learning. The review found that there is a need for interprofessional feedback training to not only address the issues in feedback but also to help learners develop a positive attitude toward the feedback process. More research needs to be performed to address learner’s perceptions of feedback providers and how to remove unwarranted biases toward out-group professions. While this scoping review includes a variety of works from the literature, more confirmatory work is needed to show how these concepts truly work in an interprofessional setting. Nevertheless, these findings contribute important information on the intricacies of feedback in interprofessional settings.

## Data Availability

No new datasets were created or analyzed in this study. Data sharing in not applicable to this article.

## Appendix A

**Table A1:** Search algorithm used in Ovid MEDLINE in October 2021.

Search Number	Search Terms
1	exp Education, Medical/
2	medical education.mp.
3	undergraduate medical education.mp.
4	student.mp.
5	1 or 2 or 3 or 4
6	exp Operations Research/
7	feedback.mp.
8	feed back.mp.
9	feedback literacy.mp.
10	6 or 7 or 8 or 9
11	exp Interprofessional Relations/
12	Interprofessional.mp.
13	Interprofessional relations.mp.
14	Inter professional
15	11 or 12 or 13 or 14
16	5 and 10 and 15
17	Limit 16 to (year = “2001–Current” and English)

**Table A2:** Data extraction from selected articles.

Study Details	Study Objective	Study Population	Methodology	Relevant Outcomes	Limitations
Tanaka et al. [14] 2017 United States	The study had three main objectives: (1) to determine what constitutes optimal feedback by conducting focus groups of anesthesia students, (2) to develop and test a web-based feedback tool and (3) to map the content of the feedback-tool’s comments	37 residents	The study used qualitative methods. Residents were invited to participate in one of five focus groups and completed written surveys that assessed their previous feedback experiences.	The focus groups revealed that there were three major barriers to good feedback: feedback that is too late to the point that it is not useful because the residents were not able to learn from feedback because it was too delayed; feedback was not specific and too general, leaving learners unable to effectively use the feedback to improve; and there were too many feedback evaluations that resulted in the feedback being unhelpful.	The study may not be generalizable to other residency institutions, as it was limited to one institution. Additionally, the study did not adequately address additional factors to the feedback process, such as fear, retention and how residents view their environment in terms of psychological safety.
Vesel et al. [15] 2016 Canada	The objective was to examine residents’ perceptions and experiences of interprofessional feedback.	131 residents (15 participated in the interviews)	The study used a mixed-methods approach. A 12-item survey about the frequency of feedback from different professionals and its value was completed by residents. After the survey, the residents were invited for a follow-up interview wherein open-ended questions about residents’ experiences, perceptions and differences with interprofessional feedback were asked. Additionally, questions about suggestions to improve interprofessional feedback were also asked in the interview.	In total, 80% of residents reported receiving written feedback only from physicians (intra-professional) and were more likely to act on said feedback. A total of 26% of residents reported receiving written feedback from nurses, and 10% reported receiving feedback from other professions. According to the qualitative results, 10 themes were found that can be broken up into three topics areas. The first topic area focused on the overall attitude toward interprofessional feedback and established that residents found interprofessional feedback to be an important element; however, they reported receiving very limited feedback from other professions that was often informal and not structured. The second topic area focused on the value of in-group versus outgroup feedback, finding that residents often preferred feedback from physicians (in-group). To the residents, physicians had a better understanding of residents’ expectations and often focused on feedback that residents perceived as more valuable. The third area focused on barriers to interprofessional feedback. Residents often found feedback to be suboptimal across all professions and not of the best quality. They also recognize that effective feedback often requires training.	Although the study included multiple residency programs, it was a single-center study, and this may limit the generalizability of the findings. The response rate to the survey was moderate, with only 52% of eligible residents completing the survey; this may cause the findings to be incomplete.
Gran et al. [16] 2016 Norway	The objective was to explore general practitioners’ (GP) and medical students’ experiences with giving and receiving supervision and feedback.	21 GPs and 9 medical students	The study was an explorative qualitative study. Two interview guides were prepared, with the one for the GPs being aimed at exploring their views and experiences with giving feedback and any potential barriers in the feedback-giving process. The interview guide for students focused on what kind of feedback they view as useful, the students’ role in the feedback process and what conditions promoted useful feedback. Two focus-group interviews were conducted for the GPs, and two focus group interviews were conducted for the students.	GPs first established mutual trust by becoming familiarized with the students’ competency levels and expectations. After this trust was established, the GPs would encourage students to be independent, while being available for supervision and feedback. The students and GPs both agreed that good feedback, which promotes learners’ professional development, was timely, constructive, encouraging and focused on ways to improve. GPs reported that giving feedback on sensitive topics such as body language, focusing on electronic devices, social or language skills, was difficult. Additionally, limited time was another barrier to giving good feedback.	Participants were recruited through the snowball-sampling method, which can cause participants with similar experiences to be included. Additionally, the members of the recruited sample were all from the same university, and this can limit the generalizability of the findings.
Van Schaik et al. [17] 2016 United States	The objective was to explore the perceptions of interprofessional peer feedback among health professionals after a team exercise.	109 medicine students (82 accessed survey and 71 completed survey), 68 pharmacy students (44 accessed survey and 41 completed survey), 59 nursing students (43 accessed survey and 40 completed survey), 49 dentistry students (25 accessed served and 24 completed survey), 16 social-work students (11 accessed survey and 9 completed survey) and 10 dietetics students (9 accessed survey and 9 completed survey)	The study was a prospective cohort study. IP learners participated in the Interprofessional Standardized Patient Exercise that was team-based and early in clinical training. After the exercise, each learner wrote anonymous feedback for each other. After giving the feedback, the students were immediately asked the level to which they agree to the statement that “Giving feedback to the students on my team from other professions was challenging”. Additionally, an online survey was completed by the learners in which they rated the usefulness and positivity of the feedback.	Overall, interprofessional learners rated giving feedback as moderately challenging. Learners found feedback to be both positive and useful. The profession of the feedback provider had no main effect. Additionally, there were no significant interactions between the professions of the feedback recipient and the feedback provider. However, there were significant effects with certain recipient professions’ rating on positivity of feedback, with certain professions (physical therapy) rating positivity higher and certain professions rating positivity lower (dental students).	The response rate was not ideal, as almost one-third of students did not access or rate the feedback they received. Additionally, the study focused on learners in the early years of their education; thus, those learners might not have had much exposure to or experience with other professions and working as a team.
Noble et al. [18] 2019 Australia	The objective was to understand the problem with student feedback literacy in the healthcare setting from the perspective of the learner.	27 healthcare students	The study used qualitative methods, using a social-constructivist approach. Learners underwent feedback-literacy programs and were subsequently interviewed to understand the learners’ perception and experiences of engaging with feedback. The interviews focused on feedback encounters.	From the framework analysis, two themes emerged: (1) the reconceptualization of feedback by learners and (2) learners’ situated engagement in workplace feedback. Following the literacy program, the students reassessed feedback as something they could engage in and not have to wait for. The students also noted that their conversations of feedback lead to plans of improvement that they could actively engage in. A challenging aspect that remained for students was being able to reconcile these new understandings of feedback in busy clinical settings.	Students’ experiences may be affected by recall accuracy. Additionally, the transferability of the findings may be limited due to the study having taken place in one hospital.
Van Hell et al. [19] 2009 Netherlands	The objective was to find empirical evidence to prove that the supervisor’s role, observation of behavior and learners’ active participation are important factors in valuable feedback.	142 medical students	The study was performed through quantitative methods. Students on the clinical rotations recorded factors surrounding each feedback event that happened to them. This included answering the following questions: Who provided the feedback? Was the feedback based on observation of behavior? Who initiated the feedback moment? What was the perceived instructiveness of the feedback?	Perceived feedback instructiveness from residents and specialists was similar but still more instructive than feedback from nurses. Feedback on directly observable behavior was reported to be more instructive than feedback on behaviors that were not directly observed. Feedback from learners’ own initiative was found to be more instructive than feedback from a supervisor’s initiative.	The number of events when feedback was given was relatively low. Furthermore, the study may be limited by demographics, particularly gender, as 78% of participants were women. Additionally, the “perceived instructiveness” variable may not represent the educational value it intended to measure due to its being based on students’ experiences.
Bowen et al. [20] 2017 United Kingdom	The objective was to explore medical students’ beliefs about feedback and how their perceptions were reflected by their feedback behaviors.	25 medical students	The study used a qualitative approach based on grounded theory. Five focus groups that contained 4–6 students each were used to analyze the learners’ perceptions of feedback in regard to the learner–educator relationship. A feedback map was used as a tool to evaluate the engagement of learners.	The three feedback behaviors that emerged are recognizing, using and seeking feedback. Influencing these behaviors are five core themes: learners’ beliefs, attitudes and perceptions; relationships; teacher attribute; mode of feedback; and learning culture. Learning culture was found to have an impact within all three feedback behaviors.	The study focused on a relatively small sample from a single institution, thus limiting generalizability.
Crommelinck and Anseel [21] 2013	The objective was to review the literature on feedback-seeking behavior in order to provide practical recommendations to educators on how to encourage such behavior.	No study population but focused on interprofessional medical settings	This study was a review of the literature (qualitative). They first defined feedback-seeking behavior and its consequences, and then they discussed various aspects and backgrounds of feedback-seeking behavior. They continued to identify issues that are unresolved in the literature. Finally, in response to the issues, they presented the self-motives framework that serves as a more comprehensive framework for understanding feedback-seeking behaviors.	Feedback-seeking behavior has inherent value in aiding in the adaption, socialization, learning, creativity and performance of learners. There are several individual and contextual factors that influence this behavior, including learning-goal orientation, public versus private environments and leadership styles. Firstly, there is higher instrumental value to feedback when oriented learners have learning goals. These learners also view failure as a way to increase effort, making them less afraid of negative feedback. Additionally, learners were found to be less likely to seek feedback when they are observed by others compared to private settings. Finally, learners tend to have higher intentions for seeking feedback from their supervisor if they view them as transformational leaders. Based on the review, the authors present six recommendations: (1) encourage learners that have low performance expectations to seek feedback so that they can learn and normalize errors and mistakes as a normal aspect of learning; (2) encourage feedback seeking during periods when there are newcomers in socialization events; (3) provide sufficient feedback; (4) train learners to develop learning-goal orientations; (5) use technology and communication to encourage feedback seeking and provide opportunities to privately seek feedback; and (6) train leaders in a variety of strategies that can encourage feedback-seeking behavior	The authors did not discuss limitations in their review of the literature. They simply discussed the findings based on a self-motives’ framework.
Teunissen et al. [22] 2009 Netherlands	The objective was to determine what sorts of variables influence feedback-seeking behaviors for residents on night shifts.	166 residents	This study was performed by using quantitative methods. Residents were sent a questionnaire that assessed four predictor variables, two mediator variables and two outcome variables. The predictor variables were learning and performance-goal orientation, and instrumental and supportive leadership. The mediator variables were perceived feedback benefits and cost. The outcome variables were frequency of feedback inquiry and monitoring.	Residents who perceived more feedback benefits were found to report a higher frequency of feedback inquiry and monitoring (taking in self-relevant information from the environment by observation of others). Additionally, those who perceive more feedback costs result in more feedback monitoring. Those who have a higher learning-goal orientation perceive more feedback benefits with lower costs. Importantly, supportive teachers (physicians) lead residents to perceive more feedback benefits and fewer costs.	Some of the variables of the study such as the “feedback benefits” variable may not be representative of the actual concept. The study also focused on one field of residents in night shifts, thus limiting generalizability.
Bok et al. [23] 2013 Netherlands	The objective was to explore the feedback-seeking behaviors of learners in a clinical setting.	14 veterinary medical students	The study was an explorative qualitative study. Semi-structured interviews were conducted with year-5 and year-6 veterinary medical students. The interview’s structure was based on the theoretical aspects of feedback-seeking behavior. The interview used questions that focused on students’ goals and motives in seeking feedback, characteristics of their feedback-seeking behavior and various factors that influence this behavior.	The interviews showed that there are personal and interpersonal factors that influence feedback-seeking behavior. Personal factors depend on the intentions of the learner, characteristics of the learner and characteristics of the feedback provider. Interpersonal factors include the relationship between the learner and the feedback provider. The analysis also showed three factors that can influence feedback-seeking behavior: ego, image and perceived benefit. Students are motivated to seek feedback that gives them positive judgments of their competence and avoid negative feedback. Those with learning-goal orientations focused on improving their knowledge and skills through feedback. The personal and interpersonal factors, as well as the potential benefits and negative effects of feedback seeking, gave rise to the feedback seeking behaviors.	The study was conducted in one setting, thus limiting generalizability. Additionally, the focus on veterinary medical education may not transfer to medical education that deals with human patients. Moreover, interview data may not capture the entire picture of feedback-seeking behavior due to the inductive method of data analysis.
Telio et al. [24] 2016 Canada	The objective was to examine how learners make credibility judgements on feedback and the consequences of those judgements.	34 residents (8 residents participated in interviews)	The study was performed by using a constructivist grounded-theory approach. Second- and third-year psychiatry residents were invited to participate. Those who responded to the invitation were asked to complete a feedback questionnaire that addressed the quality and quantity of feedback they received in clinical settings. Additionally, an Educational Alliance Scale (EAS) that focused on the relationship between the resident and the supervisor was also administered. Those with diverse scores were invited for the interviews. Semi-structured interviews were conducted for the eligible participants. The interview data were collected and analyzed to identify themes.	Participants explained that they actively contemplated feedback. They actively considered the feedback they received based on their judgments of their supervisor and their relationship with their supervisor. They were found to make judgements regularly about the supervisor’s clinical credibility during their feedback interactions, as well as the supervisor’s credibility in the education alliance. These judgements resulted in broad-ranging implications that affected the content of the feedback and their future interactions with the supervisor.	The sampling strategy, a limited number of participants and a focus on psychiatry residents may have affected the validity of the results.
Feller and Berendonk [25] 2020 Switzerland	The objective was to explore the perspectives of interprofessional feedback in the context of workplace-based assessment.	7 residents, 7 supervising physicians, and 9 allied healthcare professionals (4 diabetes nurses, 3 nutritionists and 2 psychologists)	The study used a qualitative approach based on grounded theory. Educational sessions on interprofessionalism were conducted and attended by participants. Next, workplace-based assessments were completed by residents under the supervision of supervising physicians and allied healthcare professionals. Feedback from both observers were provided after each assessment. Finally, focus-group discussions with all participants were conducted for data collection.	Four key themes were found: identity and hierarchy; interdependence of feedback source and feedback content; impact on collaboration and patient care; and logistical and organizational requirements. Feedback perceptions on interprofessional sources helped raise awareness of working conditions of other fields and helped improve communication between different groups. Perceptions on intra-professional feedback led to feedback being perceived in a more summative nature. Trustworthiness was viewed as an important factor in the feedback process, more so than professional affiliations.	The study was conducted in a single clinic and had a relatively small sample size, which may not make the results representative. Additionally, anonymity in the study may not have been properly achieved, leading to the modifications of answers.
Andrews et al. [26] 2019 Canada	The objective was to explore the perceived value of feedback by dental students on their performance on simulations by either a peer or faculty member.	126 dental students	The study used quantitative methods. Prior to the study, participants completed an interprofessional education curriculum and an Objective Structured Clinical Examination. Participants were separated into two cohorts, one that was trained in giving feedback and one that was not trained. They were then randomized into two groups: the peer-led group and the faculty-led group. In the groups, pairs viewed a video recording of the student performance in the simulation and responded to a set of reflection questions. The student was instructed to reflect on the question first and then receive input from the faculty or peer evaluator. After the feedback sessions, the groups completed a questionnaire to evaluate their experience.	In both cohorts, students valued the feedback and believed that it improved their skills. The results indicated that learners perceived clear value in participating in peer-led feedback. Despite this, students rated faculty feedback higher than peer feedback. Additionally, the learners in the peer-led group indicated that they found inherent value in the process of giving feedback, as it was a learning experience for them. This value did not differ by gender, age, class year or OSCE performance.	The study took place in a single school, thus limiting generalizability. Additionally, the lack of monitoring on a peer-led feedback group means that there is no way of knowing if students adhered to the instructions. Feedback sessions were not performed immediately after the simulation, and there was a difference in timings between when the peer group had its feedback sessions and when the faculty group had its sessions. This may affect the quality of the feedback received.
Brinkman et al. [27] 2007 United States	The objective was to determine if residents’ performance in communication and professional behaviors improved after augmenting the standard feedback on residents’ performance with multisource feedback.	36 pediatric residents	The study was a randomized control trial. Residents were randomized into either the multisource feedback group or the control group. In the multisource feedback group, residents completed a self-assessment and received a feedback report about parent and nurse evaluations. They also participated in a coaching session in addition to the standard feedback. The control group received only standard feedback. Both groups were evaluated at baseline and after 5 months, where their communication and professional skills were rated by parents and nurses of pediatric patients during the residents’ pediatric rotations.	Baseline characteristics and ratings were similar in nature. For parents, ratings increased for the multisource feedback group compared to the control group; however, the difference was not significant. For nurses, ratings increased for the multisource feedback group but decreased for the control group, with the difference being significant.	The generalizability of the study may be limited due to the focus on one institution. Additionally, the study may be underpowered to determine significant differences on parent ratings. Residents’ awareness of the evaluations may have led to the Hawthorne effect.
Van Schaik et al. [28] 2016 United States	The objective was to explore the perceptions of feedback outside of nurses and resident physician’s professional groups.	25 residents and 25 nurses (20 completed the survey)	The study was a prospective cohort study. A simulated team exercise was undertaken in which pairs of nurses and residents wrote anonymous performance feedback for each participant. Participants were then given a survey where they were asked to rate the feedback on its usefulness, positivity and agreement. Additionally, the participants were randomized into two groups. One group had four feedback comments, with two of the comments labelled with the correct profession that supplied the feedback and the other two labelled incorrectly. The other group had no labels and were asked to guess the profession of the comment’s source. Interactions between the recipient’s profession, the actual feedback provider’s profession and the perceived provider profession were examined.	There was no significant interaction between the actual profession of the feedback provider and the profession of the recipient, meaning that there was no evidence that nurses or physicians gave different ratings depending on the actual professions of the feedback providers. However, the labelling of the feedback was found to have an influence on the ratings, as there were significant interactions between the labelled profession of the feedback provider and the feedback recipient. This means that nurses rated nurse-sourced feedback higher than physician-sourced feedback, and residents rated physician-sourced feedback higher than nurse-sourced feedback. Additionally, there were significant interactions between the guessed source of feedback and the feedback recipient. Nurses who guessed the feedback came from nurses rated that feedback higher than feedback that was guessed to come from physicians. Similarly, physicians who guessed that the feedback source came from physicians rated the feedback higher than feedback that was guessed to come from nurses.	The study focused only on written feedback, and this could limit the transferability of their findings to other areas of feedback. Additionally, it was a single-center study, which can limit transferability. The study did not consider other demographic factors, such as gender and age, and this could affect feedback. Moreover, it is possible that the written feedback which was provided is not representative of the actual feedback provided in the workplace, outside of the study.
Miles et al. [29] 2020 Canada	The objective was to explore the perceptions of feedback providers and learners in order to understand how interprofessional feedback is formed, delivered, received and used.	17 residents, 11 rehab therapists, 8 social workers, 16 nurses, and 9 pharmacists	The study used the constructivist grounded-theory methodology. Individual interviews were conducted with residents, and focus groups were conducted with the other health professionals. From this, themes were identified.	The themes that captured how residents perceived feedback from health professionals and how these potential feedback providers perceive their role are as follows: the conceptualization and content of the feedback were dependent on the interprofessional relationship; perceptions of professional identity are what shape the validity of feedback; and the enactment of feedback is influenced by power and hierarchy. Feedback was found to be influenced by power differences, and residents were found to value feedback from physicians more so than from other health professionals.	The study was limited to only one institution, thus limiting the generalizability of the findings.
